# The molecular scale mechanism of deposition ice nucleation on silver iodide†

**DOI:** 10.1039/d3ea00140g

**Published:** 2023-12-21

**Authors:** Golnaz Roudsari, Mária Lbadaoui-Darvas, André Welti, Athanasios Nenes, Ari Laaksonen

**Affiliations:** a Finnish Meteorological Institute FI-00101 Helsinki Finland ari.laaksonen@fmi.fi; b Laboratory of Atmospheric Processes and their Impacts, ENAC, Ecole Polytechnique Fédérale de Lausanne Lausanne Switzerland maria.lbadaoui-darvas@epfl.ch; c Institute of Chemical Engineering Sciences, Foundation for Research and Technology Hellas (FORTH/ICE-HT) 26504 Patras Greece; d Department of Applied Physics, University of Eastern Finland Kuopio 70211 Finland

## Abstract

Heterogeneous ice nucleation is a ubiquitous process in the natural and built environment. Deposition ice nucleation, *i.e.* heterogeneous ice nucleation that – according to the traditional view – occurs in a subsaturated water vapor environment and in the absence of supercooled water on the solid, ice-forming surface, is among the most important ice formation processes in high-altitude cirrus and mixed-phase clouds. Despite its importance, very little is known about the mechanism of deposition ice nucleation at the microscopic level. This study puts forward an adsorption-based mechanism for deposition ice nucleation through results from a combination of atomistic simulations, experiments and theoretical modelling. One of the most potent laboratory surrogates of ice nucleating particles, silver iodide, is used as a substrate for the simulations. We find that water initially adsorbs in clusters which merge and grow over time to form layers of supercooled water. Ice nucleation on silver iodide requires at minimum the adsorption of 4 molecular layers of water. Guided by the simulations we propose the following fundamental freezing steps: (1) Water molecules adsorb on the surface, forming nanodroplets. (2) The supercooled water nanodroplets merge into a continuous multilayer when they grow to about 3 molecular layers thick. (3) The layer continues to grow until the critical thickness for freezing is reached. (4) The critical ice cluster continues to grow.

## Abstract 2

Schematic of the proposed deposition ice nucleation mechanism on AgI (0001).

## Graphical abstract

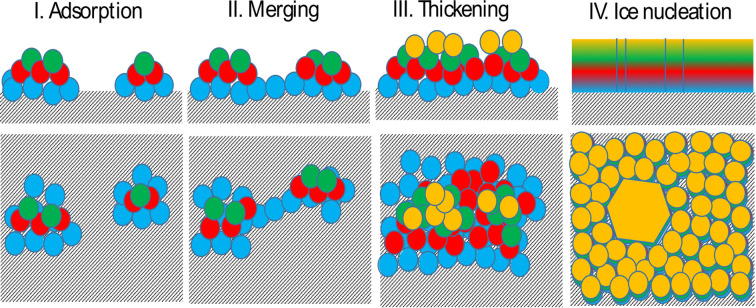

**Environmental significance**

Ice crystal formation on aerosol particles determines the optical and physical properties of cirrus and mixed-phase clouds, thereby influencing both the formation of precipitation and the effect of these clouds on the Earth's radiation budget. Deposition nucleation is one of the most frequent mechanisms for ice formation in very cold, high-altitude clouds. Despite its importance, the microscopic details of the mechanism are still largely undetermined. In this study, we investigate the mechanism on the surface of silver iodide in atomistic simulations and suggest several fundamental steps that constitute deposition ice nucleation. Silver iodide aerosol is used worldwide for cloud seeding to influence the formation of precipitation.

## 1 Introduction

Ice nucleation (IN) is a ubiquitous process. In clouds, ice nucleation determines cloud microphysical properties that in turn influence cloud radiative properties and precipitation.^1^ IN can take place homogeneously (below −36 °C),^2^ or heterogeneously, at higher temperatures or in subsaturated conditions catalyzed by the presence of an IN active surface.^3^ The leading pathway of primary ice formation in mixed phase and cirrus clouds and even clouds for which homogeneous freezing occurs can be strongly influenced by water vapor competition from IN.^4^ Heterogeneous ice nucleation occurs in three major modes:^5–7^ immersion, contact or deposition ice nucleation, depending on the environment and surface properties.

One of the least well-understood among the above is deposition ice nucleation, where according to the established description ice nucleates from water vapor directly on solid surfaces, without the presence of macroscopic amounts of liquid water preceding ice formation.^7^ Deposition ice nucleation has been studied experimentally on several natural samples and model substrates, such as clay minerals, desert dust, soot, bacteria, solid ammonium sulfate, and silver iodide (AgI).^8^ The impacts of temperature, supersaturation^8^ and particle size^9^ have been investigated. Despite all these efforts, the mechanism of deposition ice nucleation has not been established on a molecular level.^10^ One reason for this lack of understanding is the limited spatial and temporal resolution of laboratory experiments that does not allow for direct investigation of molecular scale surface properties that enhance or suppress ice formation. Molecular simulations at atomistic or coarse grained resolution have been successful in unraveling the often complex impact of surface properties on IN activity in the immersion mode.^3^ A main player in determining the formation of critical ice embryos (ice-like clusters from which ice starts to grow) is the order and binding of water molecules to the surface.^11,12^ The formation of critical embryos is affected by the properties of the solid surface such as lattice match to ice, hydrophobicity, roughness, defects, chemical impurities and porosity of the ice nucleating particle surface. A large number of molecular dynamics-based studies addressed the impact of these properties on immersion freezing on surfaces like feldspar, carbon, kaolinite, AgI or model lattices.^11,13–26^ These simulations revealed a connection between the formation of subcritical ice clusters and the preordering of liquid water to the kinetics of ice nucleation as well as to the formation of crystalline polymorphs.^12,27,28^ Most published simulation studies were performed in immersion freezing mode implying the presence of a macroscopic interface between the ice nucleating surface and water, *i.e.* the surface is embedded in water. Deposition ice nucleation – according to the current definition – does not necessarily require the presence of such a macroscopic interface, therefore one cannot draw direct conclusions on how substrate properties promote ice nucleation in the deposition mode from immersion freezing simulations. In turn, simulating deposition ice nucleation at an atomistic scale is challenging for two major reasons: to model the interaction between the vapor phase and a surface at a given water vapor pressure, *i.e.*, saturation, a specific concentration of molecules is required, which is computationally only possible applying Grand Canonical Monte Carlo (GCMC) simulations.^29^ However, the onset of crystallization can be biased by the stochastic acceptance criteria of the Monte Carlo method,^29^ and the time evolution of ice growth cannot be followed using fully stochastic sampling. Therefore, reliable crystallization can only be achieved if the movement of the molecules is deterministic and driven by real dynamics and not by probabilistic sampling. To tackle the above complexity, we adapted a hybrid Grand Canonical Monte Carlo/Molecular Dynamics (GCMC/MD) method which so far has only been used to study deposition ice nucleation in a handful of very recent studies.^25,26^

Our model substrate is silver iodide (AgI), a conventionally used cloud seeding agent and one of the best investigated inorganic ice nucleating substances, as it has been recognized as one of the most potent.^30^ Several experimental^31,32^ and computer simulation studies^19,33,34^ have investigated ice nucleation on AgI surfaces, but none of them related interfacial water adsorption to the deposition IN activity of AgI. Deposition mode ice nucleation on AgI has also been studied by environmental scanning electron microscopy,^35^ proton spin resonance spectroscopy,^36^ and X-ray adsorption spectroscopy.^37^ X-ray adsorption spectroscopy indicated that the ratio of weak and strong hydrogen bonds of adsorbed water on AgI resembles that of ice much more than that of liquid water, but the complete mechanism of deposition ice nucleation has yet to be established. Developing such mechanistic understanding is crucial for the long-needed modernization of the theoretical modeling framework to describe heterogeneous ice nucleation for atmospheric model simulations,^26,38,39^ which to date rely on the almost the century-old classical nucleation theory, with known significant shortcomings.^26,40^ In this study, we use GCMC/MD and MD simulations to investigate deposition ice nucleation on AgI surfaces at the molecular level and we suggest a comprehensive deposition ice nucleation mechanism based on adsorption theory, which is consistent with experimental data and model calculations.

## 2 Methods

To study the adsorption and freezing of water molecules on the AgI surface, we performed a Grand Canonical Monte Carlo (μVT)/molecular dynamics (GCMC/MD) simulation using the LAMMPS program package.^41^ Additionally, MD simulations were conducted under canonical ensemble (NVT) conditions to study ice nucleation on the AgI surface with different water slab thicknesses using GROMACS software 2021.^42,43^ We employ the all-atom TIP4P/Ice model^44^ in our study. This model has four interaction sites with rigid geometry and successfully reproduces the properties of bulk liquid water. It accurately predicts the melting point and coexistence curve between water and ice I.^45^ The interactions between silver Ag^+^ and iodine I^−^ ions and TIP4P/Ice water molecules are described by a Hale and Kiefer potential.^46^ Ions carry a point charge and interact with the oxygen (O) atom of water using the Lennard–Jones potential, with parameters *ε*_Ag–O_ = 0.547 kcal mol^−1^, *σ*_Ag–O_ = 3.17 Å, *ε*_I–OW_ = 0.622 kcal mol^−1^, *σ*_I–O_ = 3.34 Å for the Ag–O and I–O interactions, respectively. Both GCMC/MD and MD simulations are conducted using the charge of ± 0.6*e*.^46^ The electrostatic interactions do not take polarization into account, since TIP4P/Ice water also has rigid point charges.^19^ We assume that the AgI surface is rigid with lattice constants *a* = 4.58 Å and *c* = 7.50 Å of an ideal wurtzite crystal since (0001)-terminated AgI slabs have intrinsic dipole moments, and none of these force fields results in stable surfaces. The dimensions of periodic simulation cells are 107.8682 Å × 102.126 Å × 220.0 Å.

### 2.1 Molecular dynamics (MD)

10 independent MD simulations are performed. For the atomistic MD simulations, the thicknesses of water slabs are 5, 10, 15, 20, 25, 30, 35 and 40 Å. The simulations are conducted at constant temperatures of 213, 223, 233, 243, and 253 K using the Nosé–Hoover thermostat.^47^ The simulation systems are initially equilibrated at 298 K and subsequently cooled to reach the desired target temperatures. The equations of motion are integrated using the leapfrog algorithm with a time step of 2 fs. The cut-off for Lennard-Jones and real space electrostatic interactions is set to 8.5 Å. The PME method^48^ is used to calculate long-range electrostatic interactions. The O–H bond length and H–O–H angle of the water molecules are constrained by using the LINCS algorithm.^49^ For the MD simulations, two AgI slabs are positioned at 20 Å distance from each other in the simulation cell such that they mirror each other in order to eliminate spurious electric fields.

### 2.2 Grand canonical Monte Carlo/molecular dynamics (GCMC/MD)

Monte Carlo steps are used to model the addition and deletion of molecules until two different target vapor pressures corresponding to 6 or 60 kPa at 253 K are established. Classical molecular dynamics is used to model atomic displacements. In the GCMC/MD simulations, various ratios of Monte Carlo insertion/deletion steps are attempted alongside a 0.5 fs integration time step in the classical molecular dynamics step. The insertion/deletion step can be performed more efficiently by the Continuous Fraction Component (CFC) Monte Carlo method. Within moves of the CFC reaction, the insertion or deletion of molecules is discretized. This is achieved by gradually activating or deactivating their interactions with the rest of the system over multiple Monte Carlo (MC) moves.^50,51^ However, in this work, we follow the same insertion/deletion method of David *et al.* and Lbadaoui-Darvas *et al.*^25,26^

The MC/MD ratios tested are 500/100, 500/200, and 500/225, all conducted for a vapor pressure of 60 kPa. The two latter ratios speed up convergence and favor crystallization.^26^ For the vapor pressure of 6 kPa, an MC/MD ratio of 5 is used. As for the GCMC/MD simulations, the Lennard–Jones and electrostatic interactions are handled just as with the MD simulations, but the shake algorithm^52^ is used to constrain the O–H bond length and H–O–H angle of the water molecules. The Nosé–Hoover^47^ thermostat is used to keep the temperature constant at 253 K. Water molecules are added and deleted according to the metropolis algorithm until a stable concentration is reached. In the simulation cell, there are two empty spaces that are in contact with a slab of AgI at the center of the simulation cell. Unless otherwise stated, all simulation results discussed below are for a vapor pressure of 60 kPa and an MC/MD ratio of 500/225. Additional simulation results are reported in the ESI.† Simulations are run at 60 kPa because the simulations described above are poorly parallelizable and, thus very slow. The increased pressure accelerated adsorption to see results within a reasonable amount of wallclock time. The method of using exaggerated pressures is an established way to enhance pressure-related processes in molecular simulations.^53^

## 3 Results and discussion

### 3.1 Simulated ice growth on AgI at different temperatures

**Fig. 1:**
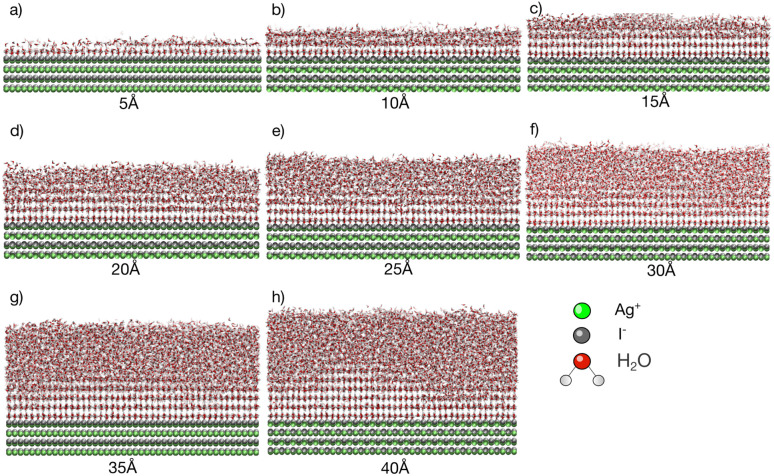
Snapshots of atomistic MD simulations of TIP4P/Ice water on AgI surface at *T* = 253 K and *t* = 60 ns. The thickness of the water slabs is (a) 5, (b) 10, (c) 15, (d) 20, (e) 25, (f) 30, (g) 35 and (h) 40 Å. Ag^+^ and I^−^ ions are colored in silver and green, respectively. TIP4P/Ice molecules are shown as red and white sticks and hydrogen bonds are shown as dashed red lines.

Simulations with water layers of varying thicknesses on the AgI surface are conducted. The simulation results illustrated in Fig. 1 show that a water slab with 5 Å thickness forms about one and a half layers of water on the AgI surface during equilibration at 298 K. As can be seen in Fig. 1a, the first layer completely covers the AgI surface, while the second layer covers it partially. For a water slab with a thickness of 10 Å, approximately two and a half layers of water molecules are formed on the surface of AgI (Fig. 1b), and finally, for water slabs larger than 15 Å, more than three layers of water are observed on the AgI surface (Fig. 1c–h).

Simulations are conducted at different temperatures, ranging from 213 to 253 K. The simulation time is 60 ns allowing us to investigate the movement of water molecules on the AgI surface. The number of ice layers on the AgI surface is determined using the ice structure recognition algorithm LICH-TEST.^54^ According to our observations, the formation of ice clusters is hindered when the water thickness is small (*i.e.*, less than 10 Å). For instance, in systems with water slabs of 5 and 10 Å thicknesses (shown in Fig. 1a and b) only two hydration layers with ice-like structures are formed. Similar results were previously reported^24^ for ice formation in slit and groove structures. They demonstrated that slit and groove geometries, which consist of only 4 layers of water, are not suitable for the formation of ice structures.

**Fig. 2:**
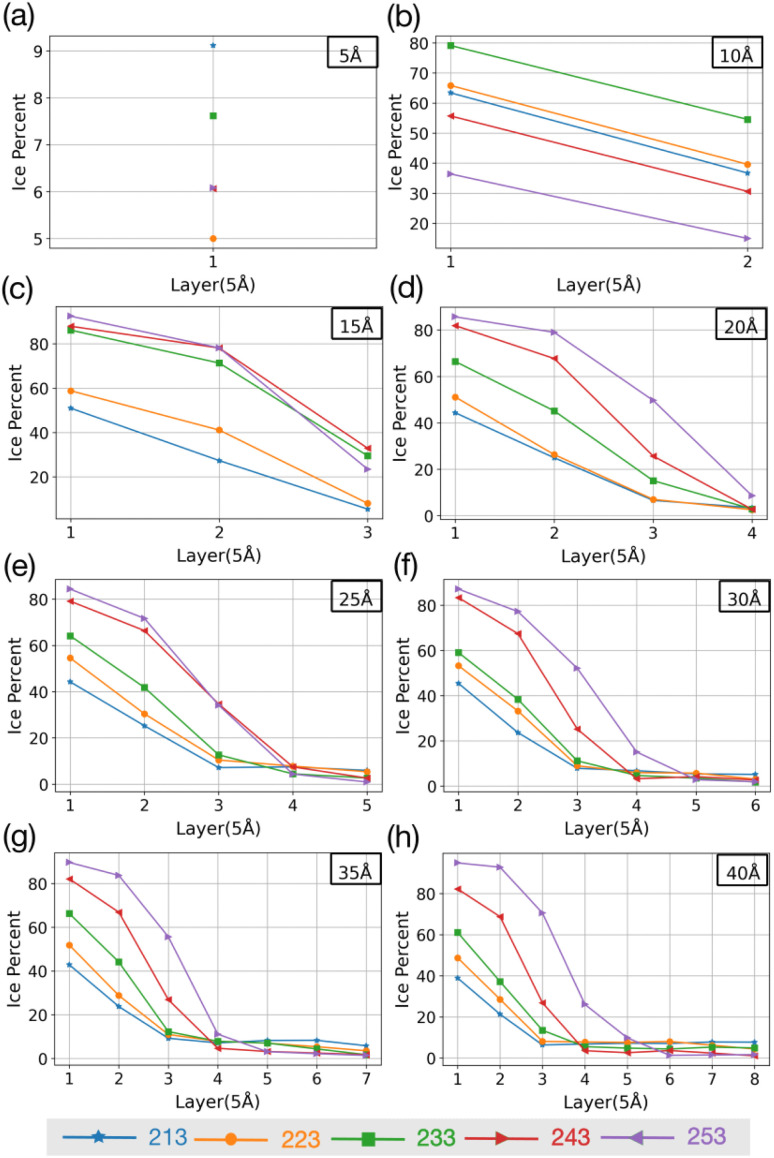
The change in percentage of ice against the distance from the AgI surface at five different temperatures. (a–h) show results for different slab thicknesses indicated in the figure.

To investigate if the embryos preferably form attached to or some distance removed from the AgI (0001) surface,^55^ we investigate the number of ice-like structures in the water slabs at different distances from the AgI surface. Specifically, the water slabs are divided into layers with 5 Å thickness, and then the ice-like structures present in each layer are determined. The results are shown in Fig. 2 and in Tables S1–S8 in the ESI.† For systems with smaller thicknesses (5 and 10 Å), we observe a larger number of ice-like structures in the first and second hydration layers at lower temperatures, specifically at 213 and 233 K, as illustrated in Fig. 2a and c. Ice number densities as a function of temperature and distance from the AgI substrate are reported in Fig. 2. Considerable ice cluster formation and ice growth are observed only in the systems with thicker water slabs, *i.e.*, thicker than 15 Å. We observe that in these systems, the number density of ice is larger in the vicinity of the substrate and at higher temperatures, especially for simulations at 243 and 253 K which resulted in over 80% ice density in the first 5 Å above the AgI substrate. The hindered ice formation at lower temperatures is a consequence of slow thermal motion hindering the spatial rearrangements needed for crystallization (Fig. 2c–h). More detailed results are provided in Tables S3–S8 in the ESI.†

The equilibrium MD simulations reveal two preconditions for ice formation. First, the simulations suggest that in order to observe ice nucleation, a minimum of four layers of water on the AgI (0001) surface is required. This corresponds to a water slab with a thickness of 20 Å. However, to achieve a proper layering of water and to obtain the correct bulk water density, a slab of water with a thickness of 25 Å is required. Second, our results demonstrate a direct relationship between temperature and the occurrence of ice structures. Specifically, we observe that as temperature increases, the number of ice structures also increases. This observation holds true throughout the duration of our simulation, highlighting the temperature-dependent nature of ice formation, and indicating that at higher temperatures ice nucleation can be promoted at higher rates. We attribute the lack of ice nucleation at low temperatures to slow thermal motion hindering rearrangements needed for crystallization to spread.

### 3.2 GCMC/MD simulations of water adsorption

**Fig. 3:**
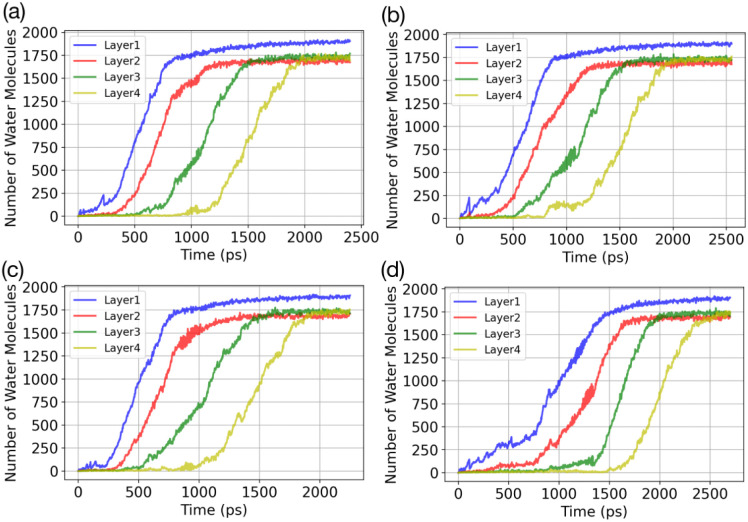
Temporal evolution of water adsorption on AgI (0001) in (a)–(d) 4 parallel simulations. The adsorbate is segmented into layers, each having a thickness of 5 Å. The vapor uptake is simulated at 253 K with *P* = 60 kPa.

Results of the GCMC/MD simulations provide detailed insights into the role of water adsorption in the deposition mechanism. We analyse our simulations using a practical approach that involves dividing the adsorbate into layers (5 Å) and monitoring the increase in the number of water molecules in each layer over time. Fig. 3 presents the temporal evolution of the number of adsorbed water molecules in the first four layers on the AgI(0001) surface. To quantify the number of adsorbed water molecules in each layer we record the time of first-layer adsorption, the quantity of adsorbed water molecules, and their surface coverage (%) for the first, second, third, and fourth layers. The results are given in Table 1. We find that successive layers form approximately simultaneously across four parallel simulations. We define the time of layer formation as the moment when the adsorbed layer shows continuous and uniform growth. The formation of the first layer is observed after 200, 305, 582, and 755 picoseconds (ps) in the four parallel simulations. The formation time of layer 2 varies randomly among the four realizations. It commences at the same simulation time as layer 1 or slightly later, but well before layer 1 is saturated. In Fig. 3a, a second layer initiates to form with a smaller number of adsorbed water molecules at the same time as the first layer. Once layer 1 contains 247 water molecules, corresponding to roughly 21.6% of surface saturation, layer 2 starts accumulating with 212 adsorbed water molecules, accounting for 18.5%. At this point, layer 3 and layer 4 consist of 36 and 18 adsorbed water molecules, respectively. Parallel simulations reveal similar numbers of adsorbed water molecules as shown in Table 1.

**Table 1:** Time until first layer adsorption, number of adsorbed water molecules and their surface coverage percentage is reported for the first (L1), second (L2), third (L3) and fourth (L4) layer at *T* = 253 K

Run	Time (ps)	N (L1)	N (L2)	N (L3)	N (L4)
a	246	137	48	20	8
		11.80%	4.10%	1.70%	0.70%
b	377	303	77	43	14
		26.48%	6.73%	3.75%	1.22%
c	743	312	111	54	25
		27.5%	9.72%	4.72%	2.20%
d	567	296	71	48	8
		25.87%	6.20%	4.10%	0.70%

**Fig. 4:**
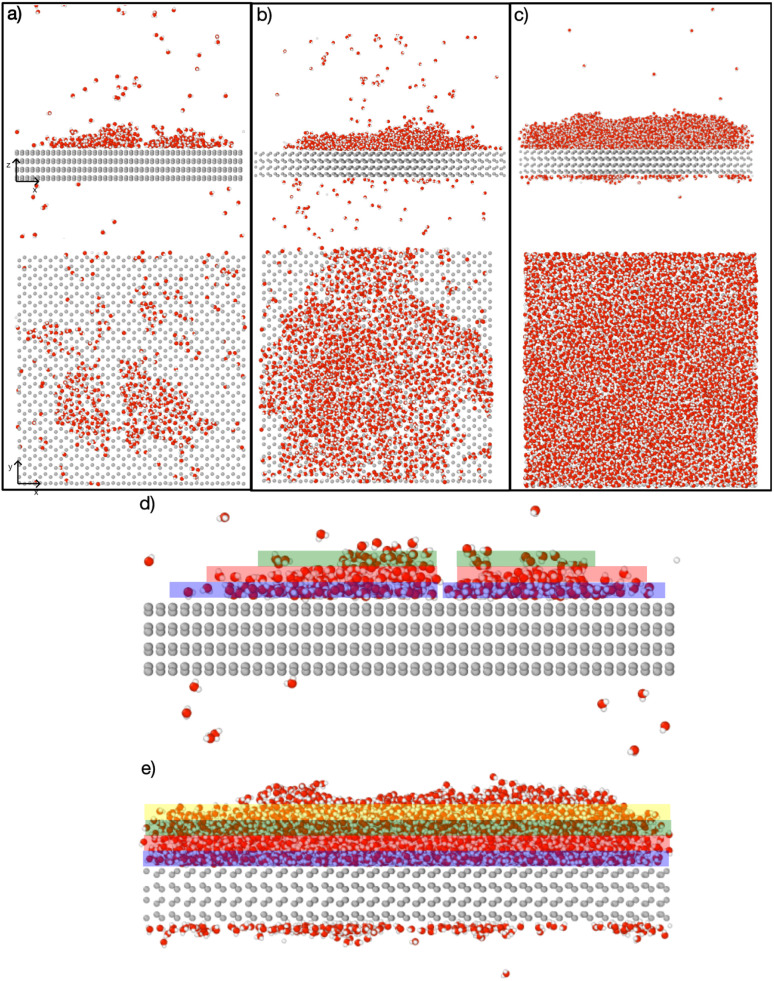
Snapshots of the temporal evolution of water adsorption on AgI (0001). (a–c) The top panel showcases a side view, while the bottom panel provides a top-down view. (a) Snapshots at 200 ps show two distinct clusters of liquid water molecules. (b) Snapshot at 570 ps reveals the merging of clusters into a single entity. (c) Snapshot at 1090 ps illustrates a shift in the adsorption mechanism from clusterwise to filmwise, leading to more uniform coverage. Figures (d) and (e) display the adsorbate at 200 ps and 1090 ps, respectively, segmented into layers, each with a thickness of 5 Å. The first, second, third, and fourth layers are color-coded blue, red, green, and yellow, respectively.

Fig. 4 depicts the adsorbed water layer build-up on the AgI surface. From the top view, it becomes evident that multilayer patches emerge initially, subsequently merging to form a layer. However, it is important to note that the formation of the multilayer occurs prior to complete surface coverage. Our observations reveal that the first and second layers start to build up concurrently in each of the simulations, marking the onset of the patchy adsorption phase. The third layer builds up with a delay in time during the patchy phase, before saturation of layers 1 and 2. The emergence of the fourth layer exhibits a considerably random nature. In three out of four parallel simulations, the fourth layer begins its formation within clustered multilayer patches, subsequently merging to build a large multilayer droplet with nearly complete surface coverage. In the fourth simulation, the multilayer patches merged before the formation of the fourth layer. The formation of liquid water as an intermediate pathway to ice nucleation has also been observed on a nonporous slab, a model of a porous slab of silica with a pore in the work of David *et al.*^25^ However, while they observed ice nucleation on the surfaces with pores, the nano-porous silica surface was unable to promote ice nucleation.

The classical nucleation theory (CNT) stands as the most widely adopted explanation for heterogeneous nucleation. CNT outlines the conditions necessary for the formation of a critical droplet or ice particle on a surface from a metastable phase, such as supersaturated vapor or supercooled liquid. It assumes a single-step process without accounting for pre-critical interactions between the surface and the vapor/liquid phase. An encouraging theoretical framework for addressing the aforementioned challenges in CNT is adsorption nucleation theory (ANT).^38,56^ By incorporating multilayer adsorption into the model, ANT is able to resolve the issue of neglecting pre-critical surface–water interactions. Our findings support ANT as we identified intermediate adsorption of liquid water and interactions between water molecules and the AgI surface, suggesting a pathway to deposition freezing.

### 3.3 Hexagonal arrangement

**Fig. 5:**
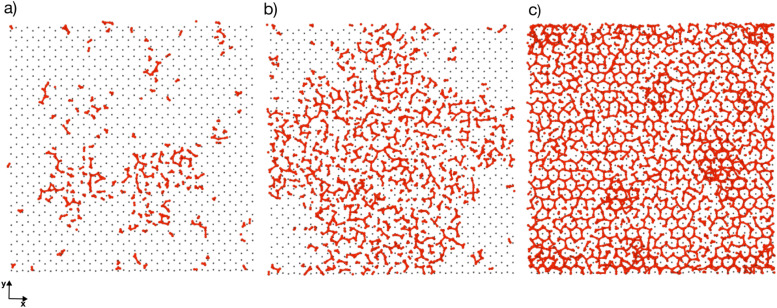
Atomistic details of the first adsorbed layer evolution on the AgI surface during the GCMC/MD simulation at (a) 250, (b) 1000 and (c) 2000 ps. AgI is colored in silver, oxygen and hydrogen atoms of the water molecules are colored in red and green, respectively.

Previous simulation studies of AgI surfaces have demonstrated that the (0001) surface terminated with silver atoms can promote ice nucleation by inducing a hexagonal pattern in the first hydration layer.^13,18,19^ This pattern resembles the basal plane of hexagonal ice (ice Ih) or the (111) plane of cubic ice (ice Ic). The GCMC/MD simulations confirm the formation of hexagonal patches in the first adsorbed layer on the AgI surface. Fig. 5a–c illustrate the build-up of hexagonal patterns over time. Initially, these patches were defective and not perfect. During the simulation trajectories, we observe that these patches appear and disappear. However, some patches grow steadily over time, and they can be clearly seen in Fig. 5b and c. The formation of a monolayer of hexagonal patches shows that the hydration layer is more ice-like than liquid-like on the surface of AgI (0001). The ice-like structures on AgI were also reported by Yang *et al.* 2021, using NEXAFS spectra of adsorbed water and the relationship to the hydrogen bonding structure of liquid water and ice at *T* = 247 K.^37^ In contrast to AgI, on TiO_2_ (ref. 57) at *T* = 235 K, the adsorbed water in the first layer was more liquid-than ice-like at different RHs. Despite the short duration of our simulations, we observe hexagonal patches in all four parallel simulations and suggest that they may serve as potential sites to initiate ice nucleation events.

**Fig. 6:**
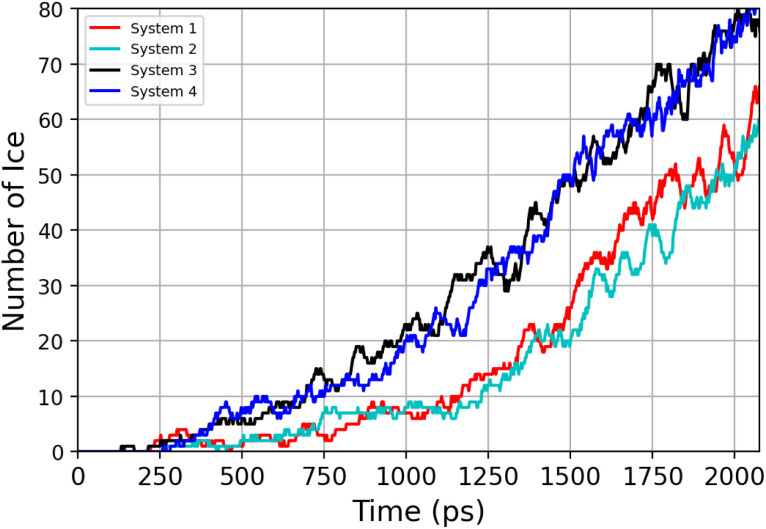
Time-evolution of the number of ice-like molecules across four parallel simulations (system 1–4).

The number of water molecules assuming ice-like structure over time for all four parallel simulations (referred to as system 1–4) is determined using the LICH-TEST algorithm^54^ (see Fig. 6). For clarity, the data shown is averaged over 10 consecutive data points. The number of ice-like molecules at the end of each simulation is relatively low with a maximum number of 80 ice molecules in systems 3 and 4. However, it can be seen that the number of ice molecules is exponentially increasing over time in all four parallel simulations.

### 3.4 Comparison with laboratory experiments

**Fig. 7:**
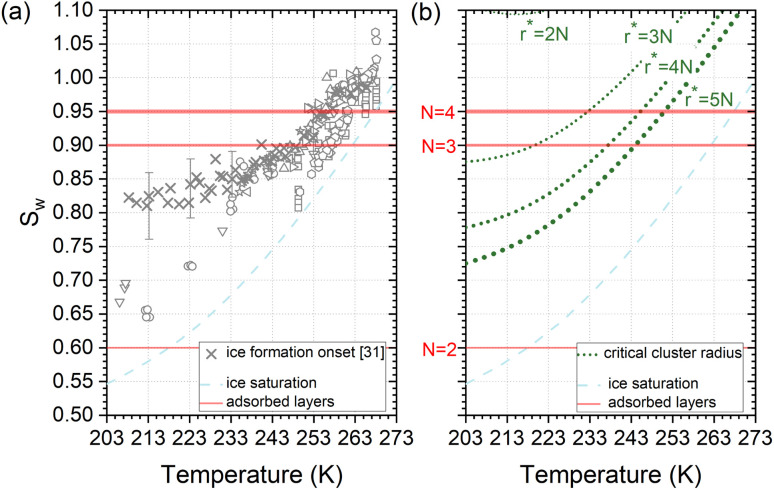
(a) Compilation of experimentally determined onset conditions (*S*_*w*_: water saturation ratio, temperature) for ice nucleation on AgI.^32^ (b) Stability conditions for critical ice embryos in water with a radius equivalent to *N* = 2, 3, 4, 5 adsorption layers. Ice saturation is shown as a dashed line, and saturation conditions for *N* = 2, 3, 4 layers of adsorption^37^ are indicated as solid lines.

Deposition mode experiments shown in Fig. 7a, suggest that ice nucleation onset conditions on AgI proceed parallel to ice saturation at *T* > 253 K but transit towards becoming parallel to water saturation at lower temperatures.

The assumption that the formation of the critical ice embryos takes place in the adsorbed water is supported by the calculations shown in Fig. 7b. The conditions at which embryos of specific size become stable are calculated by solving the Kelvin equation for spherical ice cluster surrounded by water, given by1
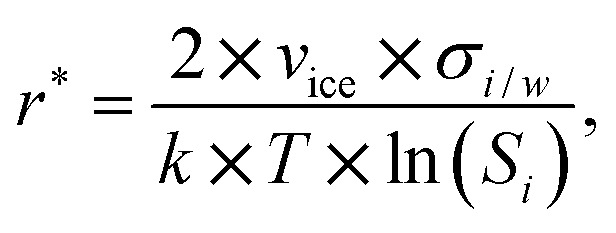
where r* is the critical ice embryo radius, *v*_ice_ is the volume of a water molecule in ice, *σ*_*i*/*w*_^58^ is the surface tension between ice and water, *k* is the Boltzmann constant, *T* is the temperature and *S*_*i*_ = *P*_*w*_/*P*_*i*_, where the *P*'s denote saturation vapor pressures of supercooled water and ice at the temperature *T*, respectively. The calculations show that the dependence of *S*_*w*_ on *T* for a fixed radius of the critical embryo is similar to the experimentally observed temperature dependence of the onset *S*_*w*_ for deposition ice nucleation on AgI particles. In addition, the simulation result (Sec. 3.1) suggests a minimum of 4 adsorbed layers to start ice formation which fits well with the agreement of the measurements in Fig. 7a and the calculation for r* = 4*N* (4 monolayers) in (b).

From the number of adsorption layers as a function of water saturation (shown as horizontal lines in Fig. 7) measured at 247 K with X-ray adsorption spectroscopy,^37^ ice formation could occur at or above *S*_*w*_ = 0.95 limited by either the formation of 4 adsorption layers (*T* < 253 K) or the critical embryo size (*T* > 253 K), respectively. Experimentally, however, ice formation is observed already at lower saturation. AgI samples most often did not undergo special pretreatment to remove already adsorbed water before experiments, which could explain some of the scatter between experiments and a deviation from the expected number of adsorbed layers. It should be noted that the precise value of the contact angle, which provides information about the ice/AgI surface free energy through the Young equation, is unknown to us. However, if the contact angle measures 90° or less, it indicates that the critical cluster aligns with the four-molecule adsorption layer.

### 3.5 Ice nucleation mechanism

**Fig. 8:**
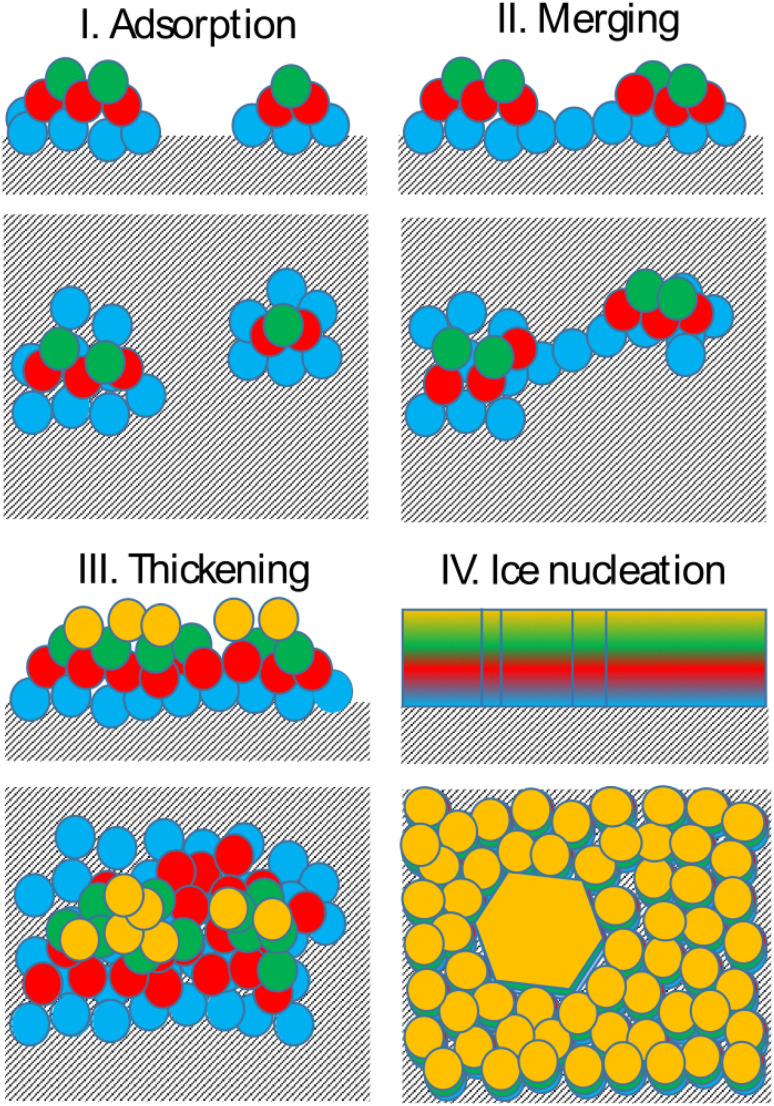
Schematic of the proposed deposition ice nucleation mechanism on AgI(0001).

Bringing together the findings presented above allows us to propose a mechanism of deposition ice nucleation on AgI, which consists of the following steps (shown schematically in Fig. 8):

1. The formation of multilayer adsorbed water patches on the surface during which the first two to three layers of water adsorbed on the surface and form hexagonally arranged islands in direct contact with the AgI surface. Hexagons in this state are imperfect and the islands appear and disappear in a random manner on the first 250 ps. The size of the adsorbed water patches as well as their spatial configuration varies from simulation to simulation.

2. Continuous multilayer formation by patch merging occurs simultaneously with the formation of the fourth layer of water on the patch. During this phase, the imperfect hexagonal network in the first layer extends, however, ice nucleation is hindered by the relatively small adsorbed layer thickness.

3. Growth of the adsorption layer until it reaches a thickness that permits ice nucleation, occurs after merging. Simultaneously the hexagonal water arrangement in the first layer stabilizes and extends.

4. Ice nucleation and growth can occur once the water molecules near the surface are ordered and the adsorption layer thickness exceeds 20 nm or about 4 layers, provided the temperature is high enough to allow molecular rearrangement of the water molecules required for crystallization within the duration of the simulation.

The results of our simulations imply that deposition nucleation only requires beforehand significant water adsorption. On AgI both the formation of adsorbed water nanodroplets and their merging are prerequisites for ice nucleation. While the timescale on which adsorption and ice nucleation follow each other is not constrained, the importance of the former cannot be neglected.

Finally, after the merging of multilayer patches, the adsorption mechanism shifted from clusterwise to filmwise. The formation of nanodroplets at lower humidities depends on the wettability of the surface. Given the hydrophobic nature of silver iodide,^31^ it suggests a tendency for droplet-wise rather than film-wise adsorption. It should be noted that whether the initial adsorption is droplet-wise or film-wise is a secondary point as the film is eventually formed on the surface. Importantly, the critical event of ice nucleation occurs within the formed film and not within the nanodroplets as we observed in our MD simulations. This emphasizes the ultimate formation of the film as the crucial factor in ice nucleation. The fact that liquid adsorption always precedes freezing challenges the classical view on deposition ice nucleation, which states that this mechanism does not involve the liquid phase at all. The liquid droplet adsorption on the surface of ice nucleating particles has been also observed in Lbadaoui-Darvas *et al.*^26^ work in which they conducted a GCMC/MD simulation on the surface of graphene.

## 4 Conclusions

A combined dataset obtained from GCMC/MD and MD simulations is used to investigate the mechanism of deposition ice nucleation on AgI.

MD simulations are performed to examine the effect of the thickness of adsorbed water films and temperature on ice nucleation on AgI(0001). The simulations are run at 213, 223, 233, 243 and 253 K with water slabs 5, 10, 15, 20, 25, 30, 35 and 40 Å thick. For slab thickness larger than 10 Å, the number of ice structures increases towards higher temperatures in all the layers. Systems with slab thickness of less than 10 Å show a higher number of ice structures at lower temperatures (213 and 223 K). We note that in the two systems with slab thickness of 5 or 10 Å, the ice structures are more interfacial than for fully developed cubic or hexagonal ice and in general the number of ice molecules is very low. Furthermore, we show that to see a perfect structure of ice, we need at least 4 layers of water molecules on the surface. This result is in good agreement with both the determination of ice nucleation onset conditions on AgI surfaces and the calculations based on r* = 4*N* (equivalent to 4 monolayers).

GCMC/MD simulations modelling the adsorption of water molecules show that water molecules adsorb in a hexagonal arrangement on the AgI(0001) surface. At the beginning of the simulations, we observe several individual patches. These patches grow in a droplet-wise manner. Later in the simulation, these small patches merge with each other and form a semi-hemisphere on the surface. Finally, we observe a shift from droplet-wise to layer-by-layer adsorption. We also quantify the number of ice-like and liquid-like water molecules in the simulation systems. In general, the number of ice-like structures is low in all of our parallel simulations, however, with a clear increase throughout the simulation time.

Based on the above results, a 4-step mechanism is proposed that starts with the formation of adsorbed water patches with thickness ranging from 2 to 3 molecular layers, followed by the merging of the patches and thickening of the adsorbed water layer until the threshold thickness needed for ice nucleation is surpassed. We conclude that deposition ice nucleation on AgI cannot happen without the preliminary adsorption of water clusters on the surface, which refines the traditional definition of the deposition ice nucleation mechanism and underlines the importance of including water adsorption into ice nucleation theories used for modelling deposition ice nucleation. These results indicate an alternative route to deposition ice nucleation involving an intermediary liquid phase. Given the significant radiative influence of cirrus clouds on climate,^59^ traditional ice formation parameterizations based on deposition ice nucleation should be substituted with models that integrate an adsorption mechanism in cirrus cloud models.^60^

## Conflicts of interest

The authors declare that they have no knowledge of competing financial interests or personal relationships that could have influenced the work reported in this paper.

## Supplementary Material

EA-004-D3EA00140G-s001

EA-004-D3EA00140G-s002

## References

[cit1] Murray B. J., O'Sullivan D., Atkinson J. D., Webb M. E. (2012). Chem. Soc. Rev..

[cit2] Herbert R. J., Murray B. J., Whale T. F., Dobbie S. J., Atkinson J. D. (2014). Atmos. Chem. Phys..

[cit3] Sosso G. C., Chen J., Cox S. J., Fitzner M., Pedevilla P., Zen A., Michaelides A. (2016). Chem. Rev..

[cit4] Barahona D., Nenes A. (2009). Atmos. Chem. Phys..

[cit5] Krämer M., Schiller C., Afchine A., Bauer R., Gensch I., Mangold A., Schlicht S., Spelten N., Sitnikov N., Borrmann S., de Reus M., Spichtinger P. (2009). Atmos. Chem. Phys..

[cit6] Cziczo D. J., Froyd K. D., Hoose C., Jensen E. J., Diao M., Zondlo M. A., Smith J. B., Twohy C. H., Murphy D. M. (2013). Science.

[cit7] Vali G., DeMott P. J., Möhler O., Whale T. F. (2015). Atmos. Chem. Phys..

[cit8] Hoose C., Möhler O. (2012). Atmos. Chem. Phys..

[cit9] Welti A., Lüönd F., Stetzer O., Lohmann U. (2009). Atmos. Chem. Phys..

[cit10] Lupi L., Kastelowitz N., Molinero V. (2014). J. Chem. Phys..

[cit11] Lupi L., Molinero V. (2014). J. Phys. Chem. A.

[cit12] Lupi L., Peters B., Molinero V. (2016). J. Chem. Phys..

[cit13] Fraux G., Doye J. P. K. (2014). J. Chem. Phys..

[cit14] Sosso G. C., Tribello G. A., Zen A., Pedevilla P., Michaelides A. (2016). J. Chem. Phys..

[cit15] Soni A., Patey G. N. (2019). J. Chem. Phys..

[cit16] Soni A., Patey G. N. (2021). J. Phys. Chem. C.

[cit17] Soni A., Patey G. N. (2022). J. Phys. Chem. C.

[cit18] Roudsari G., Reischl B., Pakarinen O. H., Vehkamäki H. (2020). J. Phys. Chem. C.

[cit19] Zielke S. A., Bertram A. K., Patey G. N. (2016). J. Phys. Chem. B.

[cit20] Ren Y., Bertram A. K., Patey G. N. (2022). J. Phys. Chem. A.

[cit21] Metya A. K., Singh J. K., Müller-Plathe F. (2016). Phys. Chem. Chem. Phys..

[cit22] Bi Y., Cao B., Li T. (2017). Nat. Commun..

[cit23] Li C., Tao R., Luo S., Gao X., Zhang K., Li Z. (2018). J. Phys. Chem. C.

[cit24] Roudsari G., Pakarinen O. H., Reischl B., Vehkamäki H. (2022). Atmos. Chem. Phys..

[cit25] David R. O., Marcolli C., Fahrni J., Qiu Y., Sirkin Y. A. P., Molinero V., Mahrt F., Brühwiler D., Lohmann U., Kanji Z. A. (2019). Proc. Natl. Acad. Sci. U. S. A..

[cit26] Lbadaoui-Darvas M., Laaksonen A., Nenes A. (2023). Atmos. Chem. Phys..

[cit27] Fitzner M., Pedevilla P., Michaelides A. (2020). Nat. Commun..

[cit28] Pedevilla P., Cox S. J., Slater B., Michaelides A. (2016). J. Phys. Chem. C.

[cit29] Lbadaoui-Darvas M., Garberoglio G., Karadima K. S., Cordeiro M. N. D. S., Nenes A., Takahama S. (2023). Mol. Simul..

[cit30] Vonnegut B. (1947). J. Appl. Phys..

[cit31] Marcolli C., Nagare B., Welti A., Lohmann U. (2016). Atmos. Chem. Phys..

[cit32] Welti A., Korhonen K., Miettinen P., Piedehierro A. A., Viisanen Y., Virtanen A., Laaksonen A. (2020). Atmos. Meas. Tech..

[cit33] Zielke S. A., Bertram A. K., Patey G. N. (2014). J. Phys. Chem. B.

[cit34] Prerna R. G., Metya A. K., Shevkunov S. V., Singh J. K. (2019). Mol. Phys..

[cit35] Zimmermann F., Ebert M., Worringen A., Schütz L., Weinbruch S. (2007). Atmos. Environ..

[cit36] Barnes G. T., Sänger R. (1961). ZAMP.

[cit37] Yang H., Boucly A., Gabathuler J. P., Bartels-Rausch T., Artiglia L., Ammann M. (2021). J. Phys. Chem. C.

[cit38] Laaksonen A., Malila J. (2016). Atmos. Chem. Phys..

[cit39] Laaksonen A., Malila J., Nenes A. (2020). Atmos. Chem. Phys..

[cit40] Zeldovich Y. B. (1943). Acta Physicochim..

[cit41] Plimpton S. (1995). J. Comput. Phys..

[cit42] Spoel D. V. D., Lindahl E., Hess B., Groenhof G., Mark A. E., Berendsen H. J. (2005). J. Comput. Chem..

[cit43] Berendsen H. J. C., van der Spoel D., van Drunen R. (1995). Comput. Phys. Commun..

[cit44] Abascal J. L. F., Vega C. (2005). J. Chem. Phys..

[cit45] Abascal J. L. F., Sanz E., Fernández R. G., Vega C. (2005). J. Chem. Phys..

[cit46] Hale B. N., Kiefer J. (1980). J. Chem. Phys..

[cit47] Nosé S. (1984). Mol. Phys..

[cit48] Essmann U., Perera L., Berkowitz M. L., Darden T., Lee H., Pedersen L. G. (1995). J. Chem. Phys..

[cit49] Hess B. (2008). J. Chem. Theory Comput..

[cit50] Shi W., Maginn E. J. (2007). J. Chem. Theory Comput..

[cit51] Moucka F., Bratko D., Luzar A. (2015). J. Chem. Phys..

[cit52] Ryckaert J.-P., Ciccotti G., Berendsen H. J. (1977). J. Comput. Phys..

[cit53] Darvas M., Hoang P. N. M., Picaud S., Sega M., Jedlovszky P. (2012). Phys. Chem. Chem. Phys..

[cit54] Roudsari G., Veshki F. G., Reischl B., Pakarinen O. H. (2021). J. Phys. Chem. B.

[cit55] Anderson D. (1967). Nature.

[cit56] Laaksonen A. (2015). J. Phys. Chem. A.

[cit57] Orlando F., Artiglia L., Yang H., Kong X., Roy K., Waldner A., Chen S., Bartels-Rausch T., Ammann M. (2019). J. Phys. Chem. Lett..

[cit58] Zobrist B., Koop T., Luo B. P., Marcolli C., Peter T. (2007). J. Phys. Chem. C.

[cit59] Matus A. V., L'Ecuyer T. S. (2017). J. Geophys. Res.: Atmos..

[cit60] Liu X., Penner J. E. (2005). Meteorol. Z..

