# Supplementary material for: The molecular scale mechanism of deposition ice nucleation on silver iodide
Source: Environ Sci Atmos. 2023 Dec 21;4(2):243–51. doi: 10.1039/d3ea00140g (PMC10867811; doi:10.1039/d3ea00140g)
Supplement: EA-004-D3EA00140G-s001 [file EA-004-D3EA00140G-s001.pdf]

Electronic Supplementary Material (ESI) for Environmental Science: Atmospheres.
This journal is © The Royal Society of Chemistry 2023

Molecular scale mechanism of deposition ice nucleation on Silver Iodide: Supporting Information

Golnaz Roudsari,[†] Mária Lbadaoui-Darvas,[*,‡,¶] André Welti,[†] Athanasios Nenes,[‡,¶] and Ari Laaksonen[†,§]

†*Finnish Meteorological Institute, FI-00101 Helsinki, Finland*

‡ *Laboratory of Atmospheric Processes and their Impacts, ENAC, Ecole Polytechnique Fédérale de Lausanne, Lausanne, Switzerland.*

¶*Institute of Chemical Engineering Sciences, Foundation for Research and Technology Hellas (FORTH/ICE-HT), 26504 Patras, Greece*

§*Department of Applied Physics, University of Eastern Finland, 70211 Kuopio, Finland*

E-mail: maria.lbadaoui-darvas@epfl.ch

The ice-like structure fraction of each layer (5 Å) of water slabs of 5 Å to 40 Å thickness for different temperatures are reported in Table S1-S8.

Table S1: Number of ice-like structures, the total number of water molecules and the ice-like structure fraction are reported for the first hydration layer (L1) on AgI (0001) surface for the system with 5 Å water slab thickness. L, I, and W denote the hydration layer, the number of ice, and the number of water, respectively.

**Water slab width (5 Å)**	**Number of ice /Number of water**
**Temperature (K)**	**L1(I/W)**
**213**	108/1141 (9.46%)
**223**	57/1140 (5.00%)
**233**	87/1142 (7.62%)
**243**	69/1138 (6.06%)
**253**	70/1135 (6.17%)

Table S2: Number of ice-like structures, the total number of water molecules and the ice-like structure fraction are reported for the first (L1) and second hydration layer (L2) on AgI (0001) surface for the system with 10 Å water slab thickness. L, I, and W denote the hydration layer, the number of ice, and the number of water, respectively.

**Water slab width (10 Å)**	**Number of ice /Number of water**	
**Temperature (K)**	**L1(I/W)**	**L2(I/W)**
**213**	725/1144 (63.37%)	598/1629 (36.71%)
**223**	753/1144 (65.82%)	642/1622 (39.58%)
**233**	905/1144 (79.11%)	882/1617 (54.54%)
**243**	637/1144 (55.68%)	501/1637 (30.60%)
**253**	416/1143 (36.39%)	247/1653 (14.94%)

Table S3: Number of ice-like structures, the total number of water molecules and the ice-like structure fraction are reported for the first (L1), second hydration layer (L2) and third hydration layer (L3) on AgI (0001) surface for the system with 15 Å water slab thickness. L, I, and W denote the hydration layer, the number of ice, and the number of water, respectively.

**Water slab width (15 Å)**	**Number of ice /Number of water**		
**Temperature (K)**	**L1(I/W)**	**L2(I/W)**	**L3(I/W)**
**213**	582/1143 (51.47%)	356/1530 (29.67%)	114/1741 (23.34%)
**223**	673/1144 (58.82%)	646/1572 (41.09%)	137/1708 (8.02%)
**233**	986/1144 (86.19%)	1103/1546 (71.35%)	511/1734 (29.02%)
**243**	1006/1144 (87.94%)	1189/1522 (78.12%)	579/1761 (32.88%)
**253**	1057/1143 (92.48%)	1196/1530 (78.17%)	408/1741 (23.43%)

Table S4: Number of ice-like structures, the total number of water molecules and the ice-like structure fraction are reported for the first (L1), second (L2), third (L3) and fourth (L4) hydration layers on AgI (0001) surface for the system with 20 Å water slab thickness. L, I, and W denote the hydration layer, the number of ice, and the number of water, respectively.

**Water slab width (20 Å)**	**Number of ice /Number of water**			
**Temperature (K)**	**L1(I/W)**	**L2(I/W)**	**L3(I/W)**	**L4(I/W)**
**213**	508/1145 (44.36%)	394/1578 (24.97%)	109/1647 (6.62%)	55/1616 (3.40%)
**223**	585/1144 (51.13%)	421/1598 (26.35%)	115/1632 (7.04%)	41/1600 (2.56%)
**233**	760/1144 (66.43%)	704/1559 (45.16%)	255/1688 (15.11%)	45/1576 (2.86%)
**243**	943/1143 (82.50%)	1046/1545 (67.70%)	440/1712 (25.70%)	42/1547 (2.71%)
**253**	980/1144 (85.66%)	1202/1522 (78.97%)	871/1754 (49.66%)	130/1515 (8.58%)

Table S5: Number of ice-like structures, the total number of water molecules and the ice-like structure fraction are reported for the first (L1), second (L2), third (L3), fourth (L4) and Fifth (L5) hydration layers on AgI (0001) surface for the system with 25 Å water slab thickness. L, I, and W denote the hydration layer, the number of ice, and the number of water, respectively.

**Water slab width (25 Å)**	**Number of ice /Number of water**				
**Temperature (K)**	**L1(I/W)**	**L2(I/W)**	**L3(I/W)**	**L4(I/W)**	**L5(I/W)**
**213**	506/1144 (44.23%)	404/1600 (25.25%)	116/1621 (7.16%)	117/1567 (7.46%)	96/1612 (5.95%)
**223**	624/1144 (54.54%)	486/1598 (30.41%)	171/1639 (10.43%)	120/1538 (8.39%)	88/1640 (5.39%)
**233**	733/1143 (64.13%)	673/1611 (41.77%)	206/1626 (12.66%)	68/1527 (4.45%)	44/1653 (2.66%)
**243**	905/1144 (79.11%)	1017/1532 (66.38%)	593/1711 (34.66%)	114/1527 (7.46%)	43/1651 (2.60%)
**253**	963/1142 (84.33%)	1089/1520 (71.64%)	592/1733 (34.16%)	68/1558 (4.36%)	15/1650 (0.90%)

Table S6: Number of ice-like structures, the total number of water molecules and the ice-like structure fraction are reported for the first (L1), second (L2), third (L3), fourth (L4), Fifth (L5) and sixth (L6) hydration layers on AgI (0001) surface for the system with 30 Å water slab thickness. L, I, and W denote the hydration layer, the number of ice, and the number of water, respectively.

**Water slab width (30 Å)**	**Number of ice /Number of water**					
**Temperature (K)**	**L1(I/W)**	**L2(I/W)**	**L3(I/W)**	**L4(I/W)**	**L5(I/W)**	**L6(I/W)**
**213**	520/1144 (45.45%)	378/1595 (23.69%)	128/1625 (7.87%)	107/1569 (6.82%)	86/1589 (5.41%)	83/1605 (5.17%)
**223**	609/1143 (53.28%)	526/1582 (33.25%)	147/1633 (9.00%)	93/1563 (5.95%)	92/1596 (5.76%)	86/1592 (5.40%)
**233**	676/1144 (59.09%)	601/1565 (38.40%)	184/1645 (11.18%)	74/1571 (4.71%)	56/1588 (3.53%)	29/1611 (1.80%)
**243**	953/1143 (83.37%)	1208/1539 (78.49%)	424/1678 (25.27%)	50/1536 (3.26)	68/1619 (4.20%)	45/1649 (2.72%)
**253**	997/1488 (67.00%)	1149/1488 (77.21%)	931/1785 (52.26%)	220/1488 (14.78%)	47/1645 (28.57%)	51/1635 (3.12%)

Table S7: Number of ice-like structures, the total number of water molecules and the ice-like structure fraction are reported for the first (L1), second (L2), third (L3), fourth (L4), Fifth (L5), sixth (L6) and seventh (L7) hydration layers on AgI (0001) surface for the system with 35 Å water slab thickness. L, I, and W denote the hydration layer, the number of ice, and the number of water, respectively.

**Water slab width (35Å)**	**Number of ice /Number of water**						
**Temperature (K)**	**L1(I/W)**	**L2(I/W)**	**L3(I/W)**	**L4(I/W)**	**L5(I/W)**	**L6(I/W)**	**L7(I/W)**
**213**	490/1144 (42.83%)	384/1616 (23.76%)	150/1622 (9.24%)	110/1540 (7.14%)	132/1600 (8.25%)	131/1575 (8.32)	93/1593 (5.84)
**223**	593/1144 (51.83%)	459/1594 (28.79%)	178/1623 (10.96%)	123/1561 (7.87%)	110/1572 (6.99%)	86/1592 (5.40%)	52/1592 (3.26%)
**233**	758/1144 (66.26%)	696/1578 (44.10%)	202/1645 (12.28%)	121/1539 (7.86%)	112/1585 (7.06%)	70/1597 (4.38%)	28/1608 (1.74%)
**243**	939/1144 (82.08%)	1009/1509 (66.86%)	459/1707 (26.88%)	72/1537 (4.68%)	52/1622 (3.21%)	41/1629 (2.51%)	28/1620 (1.72%)
**253**	1025/1144 (89.59%)	1236/1477 (83.68%)	990/1780 (55.62%)	165/1486 (11.10%)	51/1635 (3.12%)	36/1623 (2.22%)	24/1682 (1.43%)

Table S8: Number of ice-like structures, the total number of water molecules and the ice-like structure fraction are reported for the first (L1), second (L2), third (L3), fourth (L4), Fifth (L5), sixth (L6), seventh (L7) and eighth (L8) hydration layers on AgI (0001) surface for the system with 40 Å water slab thickness. L, I, and W denote the hydration layer, the number of ice, and the number of water, respectively.

Water slab width (40Å)								
**Temperature (K)**	**L1(I/W)**	**L2(I/W)**	**L3(I/W)**	**L4(I/W)**	**L5(I/W)**	**L6(I/W)**	**L7(I/W)**	**L8(I/W)**
**213**	443/1144 (38.72%)	337/1583 (21.28%)	103/1629 (6.32%)	107/1578 (6.78%)	115/1603 (7.17%)	112/1586 (7.06%)	122/1588 (7.68%)	125/1626 (7.68%)
**223**	557/1143 (48.73%)	458/1605 (28.53%)	130/1606 (8.09%)	122/1573 (7.75%)	119/1573 (7.57%)	127/1585 (8.01%)	99/1595 (8.21%)	70/1609 (4.35%)
**233**	699/1144 (61.10%)	583/1568 (37.18%)	218/1621 (13.45%)	86/1562 (4.78%)	78/1630 (4.38%)	69/1576 (4.38%)	83/1573 (5.27%)	79/1621 (4.87)
**243**	940/1143 (82.24%)	1063/1546 (68.76%)	447/1666 (26.83%)	55/1548 (3.55%)	41/1608 (2.55%)	58/1604 (3.62%)	38/1604 (2.37%)	17/1663 (1.03)
**253**	1084/1142 (94.92%)	1401/1509 (92.84%)	1273/1805 (70.53%)	362/1389 (26.06%)	161/1650 (9.75%)	21/1627 (1.30%)	24/1633 (1.47%)	27/1658 (1.62%)

We performed four parallel GCMC/MD simulations of 6 kPa vapor pressure at 253 K. The number of water molecules in the first adsorbed layer is higher than in the second, third and fourth layers. However, the number of water molecules in the first layer was relatively low, and the highest count we observed in this layer over a period of 600 ps was 35 (as can be seen in Figure S1). We estimated to see the convergence to the mentioned target pressure, a substantial amount of time was required due to the limited parallelizability of GCMC/MD simulations. Therefore, we did not continue the simulation for the vapor pressure of 6 kPa.

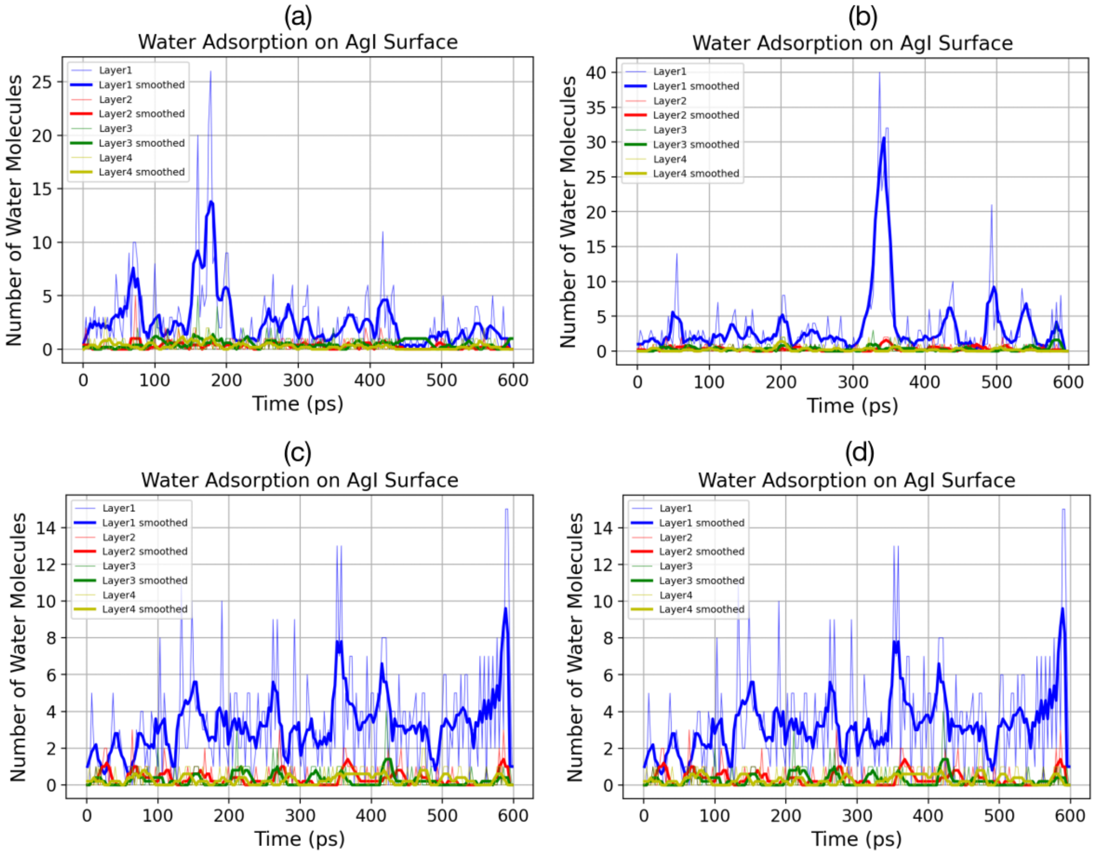

Figure S1: The temporal evolution of water adsorption on AgI (0001). The adsorbate is segmented into layers, each having a width of 5 Å. The vapor uptake is performed at 253K with 6 kPa.

At a vapor pressure of 60 kPa, four separate simulations were performed for each MC/MD ratio of 500 to 100 and 500 to 200, all conducted at a temperature of 253 K. Figure S2 shows the fluctuation in entire simulation trajectories in all four realizations. The highest amount

of water molecules in this ratio was about 460 water molecules within 10 ns. We did not observe any stable adsorbed layer for this ratio. Figure S3 illustrates the ratio of MC/MD 2.5 exhibiting similar behavior to the ratio of 2.2. However, in these four parallel simulations, there was a slight delay in the average convergence time to the target pressure compared to the ratio of 2.2. The number of ice in this ratio is also identified and the results indicate an upward trend in two out of four realizations as can be seen in Figure S4.

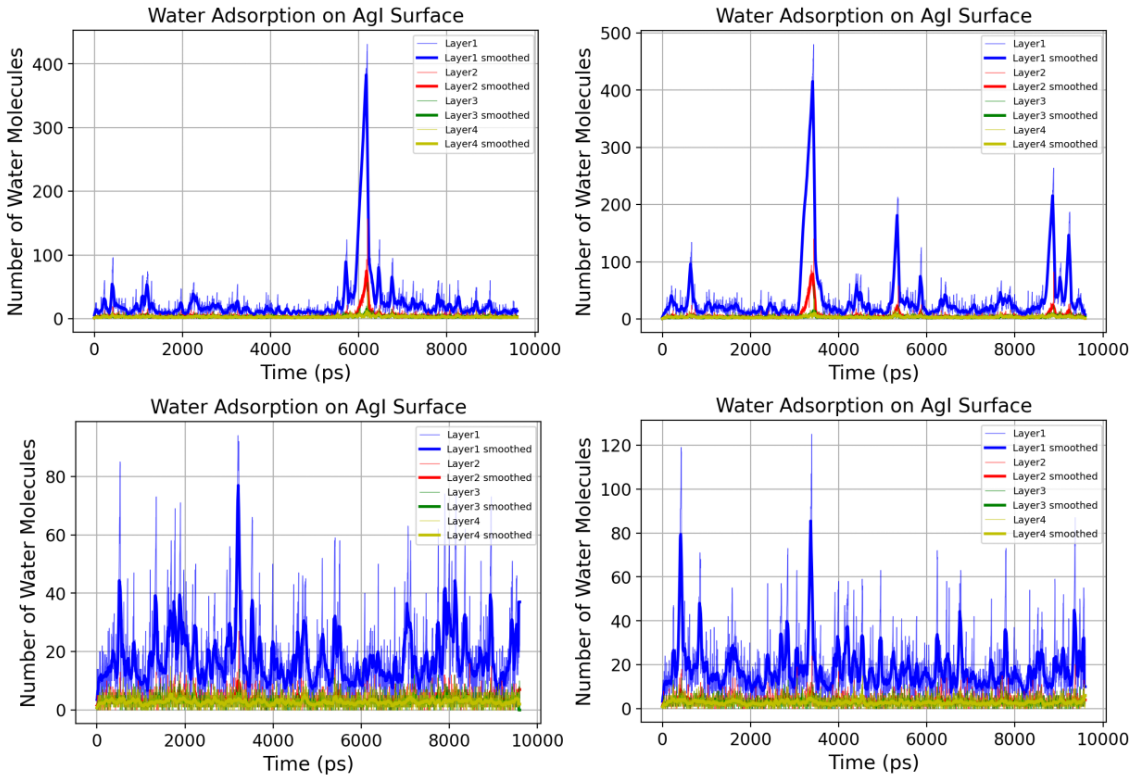

Figure S2: Temporal evolution of water adsorption on AgI (0001) in 4 parellel simulations. The adsorbate is segmented into layers, each having a thickness of 5 Å. The vapor uptake is simulated at 253 K with P= 60 kPa.

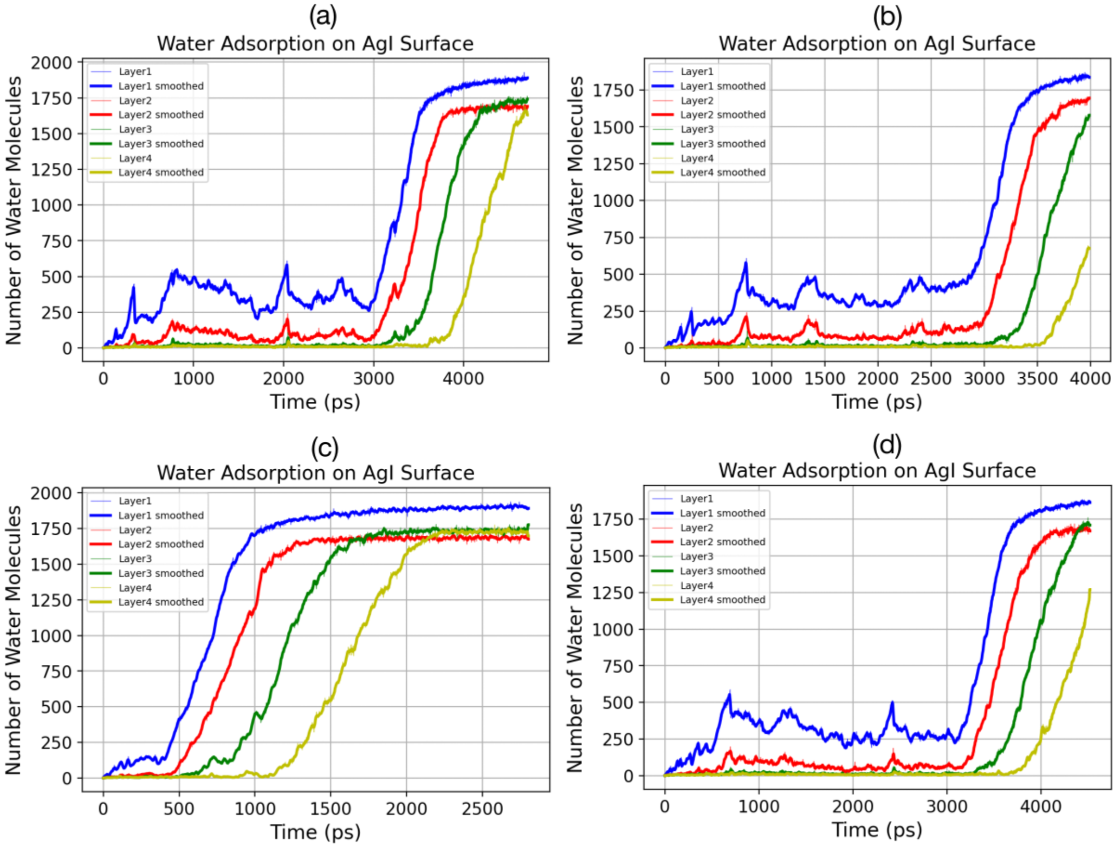

Figure S3: Temporal evolution of water adsorption on AgI (0001) in 4 parellel simulations. The adsorbate is segmented into layers, each having a thickness of 5 Å. The vapor uptake is simulated at 253 K with P= 60 kPa.

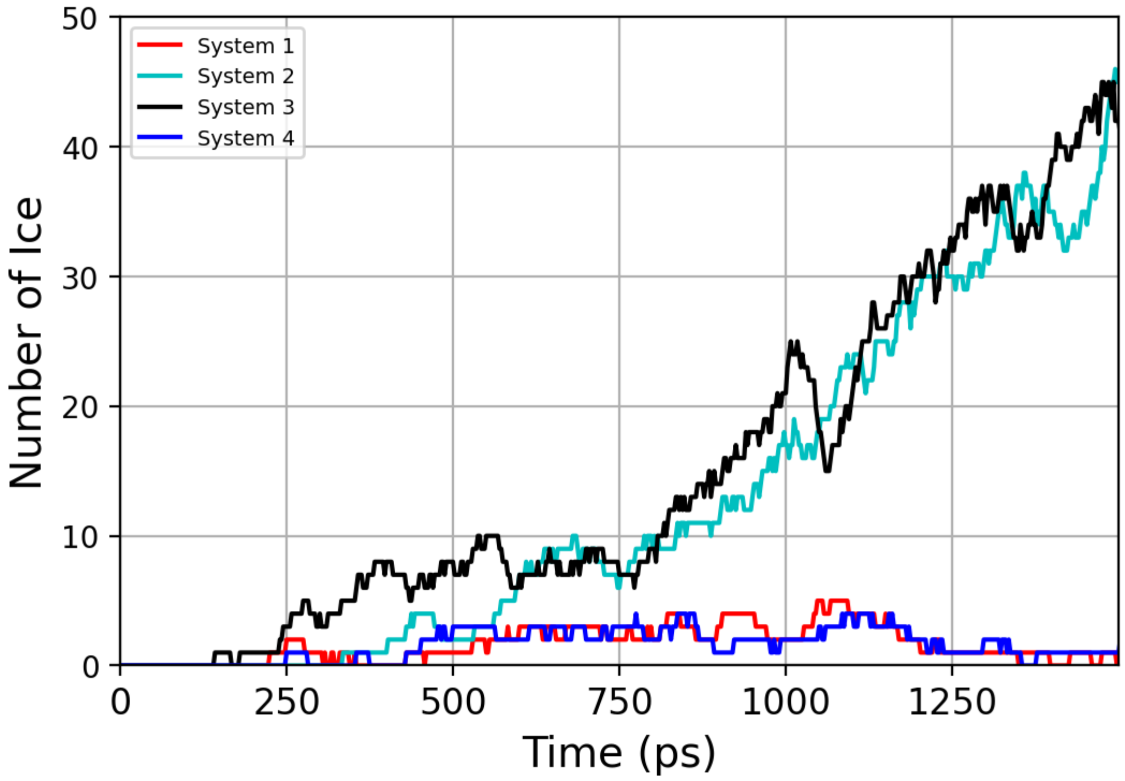

Figure S4: Evolution of ice structures across four parallel simulations throughout time.
